# Mirroring and beyond: coupled dynamics as a generalized framework for modelling social interactions

**DOI:** 10.1098/rstb.2015.0366

**Published:** 2016-05-05

**Authors:** Uri Hasson, Chris D. Frith

**Affiliations:** 1 Department of Psychology and the Neuroscience Institute, Princeton University, NJ 08544-1010, USA; 2 Wellcome Trust Centre for Neuroimaging, University College London, 12 Queen Square, London WC1N 3BG, UK; 3 Institute of Philosophy, School of Advanced Studies, University of London, Senate House, Malet Street, London WC1E 7HU, UK

**Keywords:** alignment, mirroring, synchronization, coupling, inter-subject correlation

## Abstract

When people observe one another, behavioural alignment can be detected at many levels, from the physical to the mental. Likewise, when people process the same highly complex stimulus sequences, such as films and stories, alignment is detected in the elicited brain activity. In early sensory areas, shared neural patterns are coupled to the low-level properties of the stimulus (shape, motion, volume, etc.), while in high-order brain areas, shared neural patterns are coupled to high-levels aspects of the stimulus, such as meaning. Successful social interactions require such alignments (both behavioural and neural), as communication cannot occur without shared understanding. However, we need to go beyond simple, symmetric (mirror) alignment once we start interacting. Interactions are dynamic processes, which involve continuous mutual adaptation, development of complementary behaviour and division of labour such as leader–follower roles. Here, we argue that interacting individuals are dynamically coupled rather than simply aligned. This broader framework for understanding interactions can encompass both processes by which behaviour and brain activity mirror each other (neural alignment), and situations in which behaviour and brain activity in one participant are coupled (but not mirrored) to the dynamics in the other participant. To apply these more sophisticated accounts of social interactions to the study of the underlying neural processes we need to develop new experimental paradigms and novel methods of data analysis

## 1. Introduction

The discovery of mirror neurons [1] that respond similarly to both performing an action and observing the same action, generated an enormous excitement in the scientific community. It was proposed that mirror neurons were involved in our ability to learn new skills by imitation [2], to understand other people's actions [3], to simulate other people's intentions [4], thoughts, and even emotions [5], and finally to be involved in language acquisition [6]. Usually, mirror neurons are defined as a set of perception/action neurons within an individual brain, which provide the necessary link between the perceptual and motor systems. Given, however, that (i) in most experimental set-ups, mirror neurons were tested in the context of a dyad, when one brain is performing the action while the other brain is perceiving it; (ii) most proposed functions attributed to mirror neurons are related to social interaction across two or more conspecific members (one acting, while the other is perceiving)—in this paper, we will discuss mirror neurons in a wider context of action/perception coupling across (rather than within) individuals. As we shall argue below, placing mirror neurons within a two-brain framework can prove a fruitful exercise for understanding the utilities as well as the limitations of the mirroring concept.

There is an inherent tension with the mirror neuron concept that was partly acknowledged by some [7–9], but was never explicitly resolved. Mirror neurons were originally discovered within the context of motor acts and were described as neurons that code a specific set of actions directed toward an object (e.g. grasping a piece of food with a precision grip). Subsequently, it was found that other classes of mirror neurons encoded motor acts at a more abstract level (e.g. grasping a piece of food even when this is achieved by different effectors or even tools, see [10] for a review). The extension of mirror neurons to the domain of intentions generates a tension between the specificity of the motor act and the broader abstract goal associated with that act: if a mirror neuron is narrowly tuned to a specific motor act (i.e. mirrors the actions) then its explanatory power is greatly diminished. By contrast, if a mirror neuron is broadly tuned to a goal (i.e. mirrors the intentions) then it may lose its motor-based grounding.

**Figure 1.**
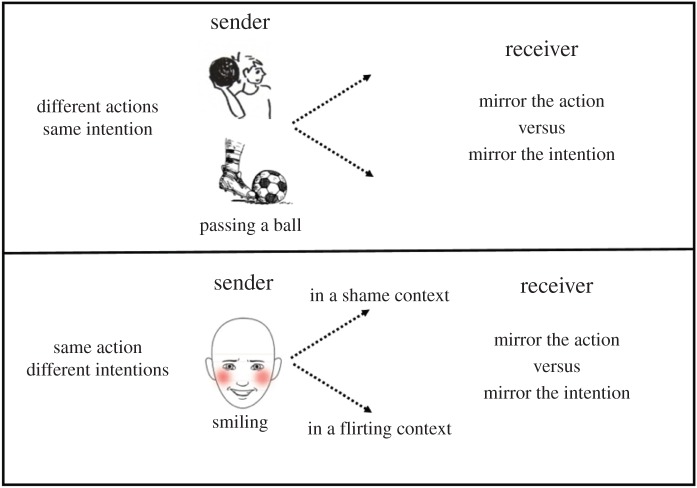
Do we mirror actions or intentions? (Online version in colour.)

To demonstrate the problem, consider two simple scenarios (figure 1). In the first scenario, the sender's intention is to pass the ball. However, in one case the sender does so by throwing the ball, whereas in the second case the sender does so by kicking the ball. If the receiver is mirroring the action, different sets of neurons will respond to kicking versus throwing. If the receiver is mirroring the intention, however, the same set of neurons will respond to both actions. In the second scenario, the sender performs the exact same act of smiling. However, in one scenario she smiles after dropping the birthday cake to the ground (shame context), while in the other scenario she smiles to signal that she likes the person she meets on a blind date (flirting context). If the receiver is mirroring the action, this will result in the same neurons responding in both contexts. If the receiver is mirroring the intentions, however, then different sets of neurons should respond in the ‘shame’ context versus the ‘flirting’ context (figure 1) [7].

These simple examples raise the question as to the level of abstraction that is being mirrored, from an exact copy of the actual behaviour, to an effector-based motor action, and up to an abstract mirroring of goals and intentions. In addition, it raises the question as to whether mirroring, when attributed to abstract goals and intentions (e.g. for passing a ball), is the appropriate terminology, given how far removed the representation is from the actual physical manifestation. In this case, we propose using the term ‘alignment’. In its most basic form, alignment is observed when birds and fish move together to create a flock or shoal. Here the movements of the individuals are physically aligned [11]. In humans, much more abstract forms of alignment can be observed when the behaviours of players are aligned through their common knowledge of the rules of a game. Alignment is essentially a form of imitation. The concept of neural mirroring provides a mechanism that underpins the various kinds of behavioural alignment that can be observed.

## 2. The importance of mirroring and alignment

The phenomenon of alignment is an extremely important aspect of social interaction. After all, interactions with other members of a group can fundamentally shape the way we behave in the world, and alignment is a ubiquitous feature of such interactions. Alignment facilitates cultural learning, maintenance of culture and group cohesion (see, e.g. [12]). One example of alignment is the automatic mimicry of postures, mannerisms and facial expressions during face-to-face interactions (the chameleon effect [13]). Experimental studies show that such mimicry increases rapport between partners [14] and increases prosocial behaviour even beyond the situation in which the mimicry occurs [15]. Further evidence for the importance of alignment for social cohesion comes from the observation that both children and adults show enhanced mimicry of their peers in the face of ostracism from their in-group [16,17].

However, as we discussed in §1, mirroring and alignment can occur at many levels from the concrete to the abstract. This is especially notable in conversations where people align their speech rate, their choice of words and, ultimately, their high-level mental representations [18]. Even low-level alignment during discourse can improve mutual understanding. For example, imitation of an unfamiliar foreign accent improves spoken language comprehension [19].

A particularly important form of high-level alignment, that is probably uniquely human, is that created by culture. This form of alignment solves coordination problems through conventions such as driving on the left [20]. Shared rules and customs also play a key role in the coordination of many other verbal and non-verbal social and cognitive skills, such as conversing, courting, dancing or even navigating a ship [21].

In this paper, we will discuss the concepts of mirroring and alignment within the broader context of a brain-to-brain dynamic coupling framework. In this context, mirrored responses between interacting participants are a specific form of dynamical coupling (i.e. synchronized coupling). However, our approach can easily encompass both action-based mirroring as well as intention-based alignment, and, most importantly, goes beyond the concept of alignment to situations in which the responses are coupled, but not mirrored across brains.

### (a) A coupled neural dynamic framework for studying neural and behavioural alignment

Placing mirroring in the wider context of sender–receiver interactions opens up new ways of viewing neural alignment across brains. In this context, the receiver is not simply a passive observer. Any interaction across a dyad can be conceived of as a dynamic process by which information is exchanged between individuals. A facial expression can transmit information about the sender's mood and a motor gesture can signal the sender's intention.

Both mirroring the motor act of a sender and mirroring the intentions of a sender can be thought of as reflecting two specific forms of coupled neural activity across a sender and a receiver. We will use the terms *neural alignment* and *mirror responses* interchangeably to denote neural responses which are correlated (i.e. similar) across brains. We will use the inclusive term *neural coupling* to denote neural processes that have lawful relationship across the sender's and receiver's brains. This can include correlated activity (neural alignment), as well as complementary actions and other lawful transformations and dynamical interactions that influence and constrain the sender's and receiver's neural responses.

Brain-to-brain coupling is mediated by sounds and bodily movements generated by the sender and picked up by the receiver. Thus, during effective interaction, the sender's brain actively and systematically influences the receiver's neural response. Lack of sender–receiver coupling indicates that information has not been successfully communicated. In early sensory and motor areas, two brains can be aligned to the specific input structure (e.g. in cases when both the sender and receiver see hand movements performing the action, the responses in high-order visual areas may be aligned across both brains)—this can be thought of as a simple low-level mirroring. In mid-level areas, the responses can be aligned to a specific act produced by a specific effector—this can be thought of as action-based mirroring. In high-order areas, the responses across the two brains can be similar only when meanings and intentions are shared across brains in a particular context—this can be thought of as intention-based mirroring. Because such intention-based responses usually depend on abstract inferences that take the contextual cues into account, such mirroring can no longer be considered as being closely aligned to the motor system.

A bottom-up, reductionist framework, which divorces perception from action and contextual meaning from individual acts, is fundamentally incomplete, so the insights that it can reveal about the nature of interaction are inherently limited. Full understanding of how and why we interact with others therefore requires shifting from a bottom-up, ‘one-brain in isolation’ to a ‘multiple-brain’ frame of reference in which interactions play a major role [22]. We also need to move from artificial laboratory conditions to natural, real-life settings [23].

### (b) Brain-to-brain coupling during verbal communication

In this section, we will discuss mirroring and alignment at different levels of abstraction. We will focus on verbal communication, as this is a natural extension of the sender–receiver coupling idea, but in a domain that allows us to establish links between production processes in the speaker's (sender) brain and comprehension processes in the listener's (receiver) brain.

Most studies of verbal communication focus either on production-based processes in the speaker's brain or on comprehension-based processes in the listener's brain, typically using highly constrained tasks (e.g. the production or comprehension of single phrases). By design, this approach precludes studying how one mind transmits information to another and ignores the larger context in which individuals interact. Thus communication, which by nature is a joint action embedded in a social context, is paradoxically studied in single individuals in isolation. In contrast with this reductionist framework, we have measured neural coupling across a speaker and a listener during real-life communication (i.e. during story telling) using inter-subject correlation analysis (ISC).

The ISC method has been previously applied to measure neural responses to naturalistic stimuli such as movies or stories; the responses in one brain are used to predict responses in another brain perceiving the same stimulus and/or engaged in the same behaviour. Because spontaneous fluctuations and noise are not correlated across brains, this measure exposes neural responses that are shared across subjects. For example, subjects watching the same movie during functional magnetic resonance imaging (fMRI) show similar neural dynamics time-locked to the stimulus [24]. This result has been extensively replicated using single-unit recordings [25] and electrocorticography [26]. Crucially, ISC can detect neural dynamics in high-order brain areas that are shared across people and associated with the speech content and not the physical form used to convey it. For example, the same neural responses were observed in high-order areas in Russian speakers listening to a story in Russian and in English speakers listening to the same story in English, suggesting that these high-order areas represent the narrative irrespective of its form [27]. Furthermore, the alignment of responses across subjects in these high-order areas is sensitive to the interpretation of the narrative content [28]. For example, providing an explanatory context can alter subjects' interpretation of a story, leading to stronger similarity among people with a shared interpretation relative to people without such an interpretation. These results demonstrate that shared activity in high-order brain areas across receivers is locked to the meaning of the input within a given context. If such responses are also seen in the sender's brain, then this is evidence for an intention-based alignment or mirroring of brain dynamics across the dyads.

**Figure 2.**
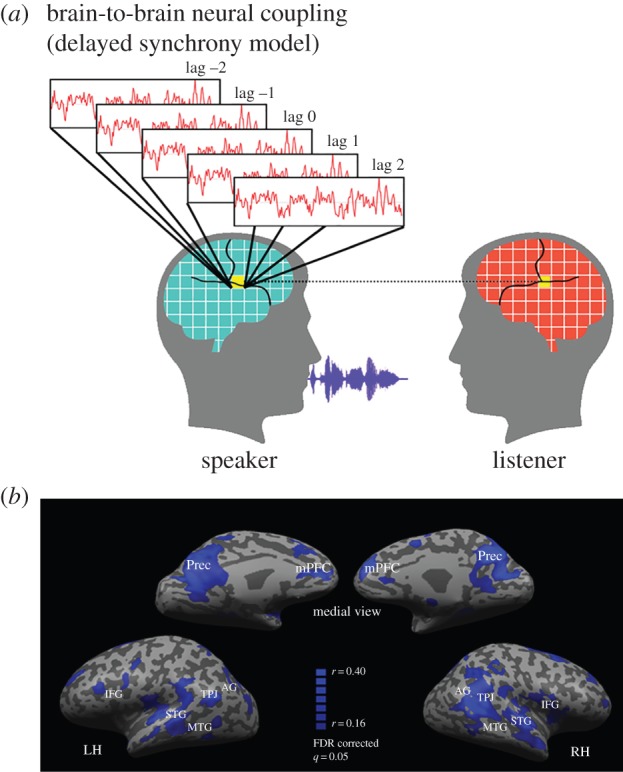
Brain regions where activity in the listener mirrors that in the speaker.

To test whether the listener's (receiver's) brain mirrors the speaker's (sender's) brain during verbal communication, we extended ISC analysis to dyads. We used the speaker's brain activity as a model to examine the listener's brain activity over time, thereby circumventing the need to specify a formal model of the neural response in any given brain area. This analysis exposes neural responses in the listener's brain that are correlated to those in the speaker's brain at a constant temporal interval (i.e. the listener's responses consistently either precede or lag behind the speaker's responses). In these studies, a single speaker tells a story while being scanned using fMRI. The speaker's story is recorded during scanning, and then played back to listeners in a subsequent session, also during fMRI. This allowed us to map brain areas in which neural responses were mirrored (aligned) across the speaker's and listener's brains (figure 2*a*). The analysis revealed that the listener's brain activity mirrors that of the speaker with a typical delay of 3 s (figure 2*b*). This mirroring (alignment) was eliminated when the communication signals were misaligned. The strength of mirroring was positively correlated with story comprehension [29,30].

These studies reveal that production-based processes in the speaker's brain are aligned with comprehension-based processes in the listener's brain (and that the stronger the alignment across brains the better the comprehension [29–31]). The speaker–listener alignment occurs at many levels. In early sensory areas, the shared neural patterns are aligned to the low-level properties of the stimulus, such as acoustic features, while in higher-order brain areas the shared neural patterns are aligned to the high-level aspects of the stimulus, such as their meaning. Such alignment is an important precursor for successful social interactions, as it provides grounding in common knowledge and expectations. Successful social interactions depend on the creation of such common ground [32]. The existence of common ground is necessary for people to experience the world in the same way and for the listener to have a chance of understanding the speaker. Indeed, the degree of alignment between the speaker's and listener's brain responses predicted the listener's comprehension level [30].

## 3. Beyond mirroring and alignment

As we argued earlier, alignment and mirroring alone are not sufficient to explain the rich and versatile ways we interact with each other. Frequently we wish to be coupled with others, without necessarily mirroring their behaviour. For example, when playing soccer, players will coordinate their moves and their responses will be coupled to the movements of other players. However, each player will perform a different set of movements to maximize the cooperation among players from the same team while interrupting the dynamic of the other team. Interactions are dynamic states which involve continuous mutual adaptation, the development of complementary behaviour, and division of labour such as leader–follower roles. Participants can be said to be coupled, rather than aligned, as such a framework can encompass both states in which the behaviour and brain activity mirror each other (neural alignment) as well as situations in which the behaviour and the brain activity in one person are coupled (but not mirrored) to the dynamics in another. Recently, there have been many proposals for characterizing such coordination dynamics (e.g. [33]). Our concern in this paper is to develop such ideas in the form of behavioural paradigms and neural methods of analysis that can be applied in the context of dyadic interaction between a sender and a receiver.

### (a) Social interaction involves mutual adaptation

**Figure 3.**
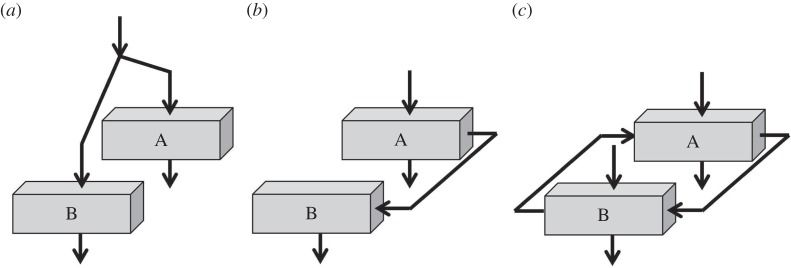
(*a*) Actors A and B are both driven by the same input. (*b*) Actor B is driven by actor A, one-way information transfer. (*c*) Actors A and B mutually exchange information.

Consider the system illustrated below (figure 3*a*). This shows two agents responding to the same external signal. For example, two people might be playing instruments in time with a metronome beat. However, they do not hear each other, only the beat of the metronome. They are acting together and their playing can be highly synchronized, but as they are not interacting, there is no exchange of information.

Another system in which people act together has exchange of information, but in one direction only (figure 3*b*). This is an extreme case of leader and follower. In this example, A is following the metronome beat, and B is following A. But A has no information about what B is doing. This is a one-way interaction because the information only flows one way. Here again the behaviour of A and B could be highly synchronized, showing that synchronized behaviour is not a necessary marker of two-way social interaction. A two-way interaction is illustrated in figure 3*c*. Here the exchange of information between A and B runs simultaneously in both directions (as in verbal dialogues).

**Figure 4.**
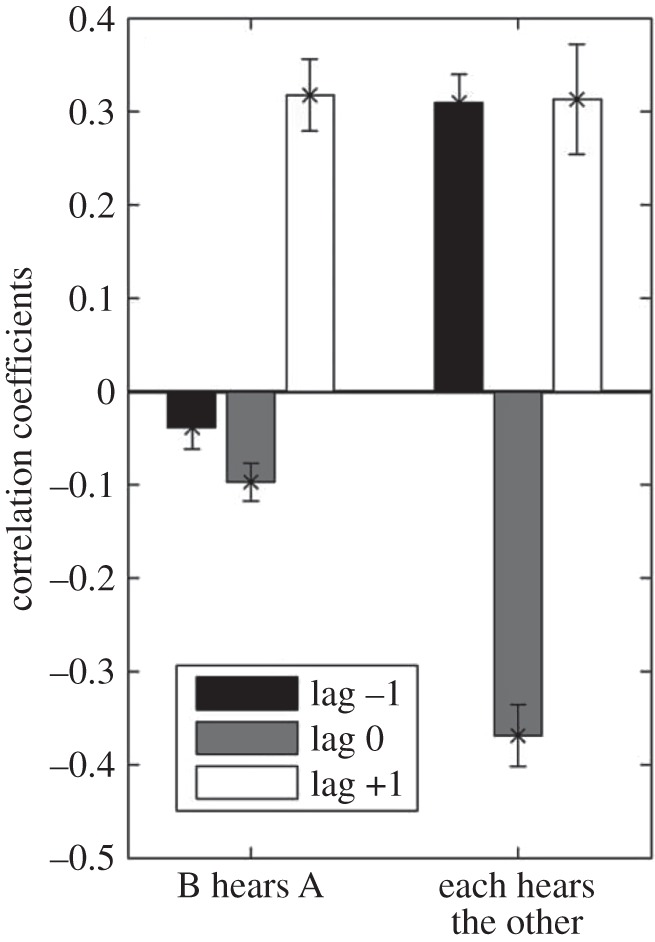
Cross correlations of inter-tap intervals in a synchronized tapping task (redrawn from Konvalinka *et al*. [34]).

An interaction with this two-way flow was studied by Ivana Konvalinka in a synchronized tapping task. Pairs of participants were instructed to maintain a particular tapping frequency while also tapping in synchrony [34]. A could hear B's taps and B could hear A's taps. One indication of synchrony in the performance of this task is that both partners have the same interval between successive taps and participants easily achieved such synchrony. However, a different picture emerged when the data were examined in more detail. This was done by looking at the moment-to-moment relationship between inter-tap intervals, by measuring their cross-correlation. At lag zero this correlation was typically negative. This is because the partners were continuously and mutually adapting to one another. So if partner A had been slightly faster than B on the previous trial, then she would slow down on the next trial. However, at the same time B would be speeding up, having been slower than A on the previous trial. At lag 1, this behaviour leads to a positive correlation as A is imitating the behaviour of B (and vice versa) on the previous trial (figure 4). In many cases, a leader–follower relationship emerges as one partner may adapt less than the other. This is revealed by the difference in correlations at lag +1 and lag −1, as lag +1 reflects how much A adapts to B, while lag −1 reflects how much B adapts to A. In this task, coupling between the partners is achieved and maintained, not through perfectly synchronized behaviour, but through continuous mutual adaptation.

**Figure 5.**
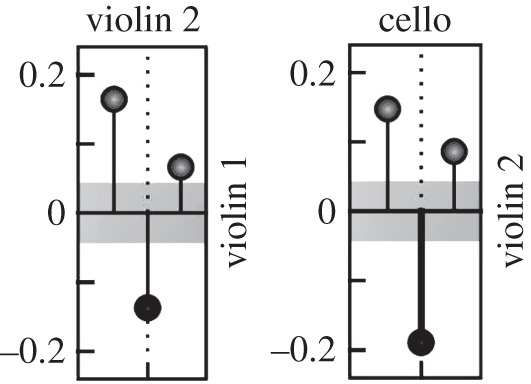
Cross correlations of inter-note intervals in a string quartet. Violin 2 is following violin 1, while the cello is following violin 2 (redrawn from Wing *et al.* [35]).

This pattern of adaptation is also observed outside the laboratory in the behaviour of the members of a string quartet. Wing *et al.* [35] applied cross-correlation to the inter-note intervals of the players. In one of the two professional quartets studied, the first violin was clearly the leader in the terms described above, as the other players adapted their playing more to her than she did to them, showing the characteristic pattern of correlations for lag +1 and lag −1 (figure 5). We suggest that it is this continuous pattern of mutual adaptation, rather than simple mirroring, that is characteristic of many real-time social interactions.

### (b) Alignment and the we-mode

The process of mutual adaption, described above, generates coupled behaviour among agents on a moment-to-moment basis. The tendency to mirror the behaviour of others can thus generate alignment among individuals in a group. For example, alignment at the most basic level underlies the spectacular flocking behaviour seen in birds, fish and many other animals [36]. The mutual alignment of the individuals in these groups is based on a few simple rules, of which the most important is to move in the same direction as your closest neighbours. This enables the group to move as an entity without the need for any centralized control [11]. Such alignment creates advantages for the group and for the individuals of which the group is composed. For example, through their mutual interactions, a shoal of fish can follow a chemical signal leading to food that would be too weak for an individual to follow [37,38] and a flock of homing pigeons can navigate better than the best individual [39].

Such automatic, spatial alignment behaviours (i.e. flocking, herding) are also observed in humans, both experimentally in the laboratory [40] and in real life. For example, a group of pedestrians crossing a busy street show spatial alignment [41]. However, humans also automatically align at many other, more abstract levels. We already mentioned that many different levels of alignment occur in dialogue. At the most concrete level, speakers mutually adapt to each other's speaking rate. At a more abstract level they imitate each other's use of words, resulting in alignment of vocabulary and syntax [18,42]. When people work together on a novel task, they spontaneously develop and align the terms they use for talking about relevant aspects of the task. If, for example, the task was to detect a weak visual signal, then it is important for people to agree on terms for describing how confident they are in what they have just seen. The phrase ‘quite confident’ must refer to the same level of confidence for both partners. Achieving such alignment of terms for talking about confidence generates an advantage, as the group can then perform better on the task than the best individual within the group [43]. This is because they can follow the lead of the more confident person on a trial-by-trial basis.

When people become aligned in this way, they can be said be in the *we-mode* [44]. They are representing the various relevant aspects of their environment in a common format. For example, when alone, a person will represent the location of an object as ‘within *my* reach’. But when in a group, objects can be represented by everyone present as ‘within *our* reach’, even when out of reach a particular individual [45]. These we-mode representations align the group's appreciation of the situation and provide a common grounding for joint actions. Such alignment has been demonstrated for many aspects of the shared environment. When people are together, they take account of each other's potential for action (affordances, [45]), each other's goals [46] and each other's knowledge [47]. These effects mostly occur automatically and without awareness (see e.g. [48]).

### (c) Achieving dynamic coordination

There are, however, many situations in which alignment can lead to disadvantages. Too much alignment can lead to the ‘folly of crowds’ or herding behaviour [49]. One example is the information cascade where people make decisions in sequence and know the decisions of the previous participants. Someone whose personal knowledge slightly favours buying stock option A, may well choose to buy option B if this has been the choice of the majority so far. This is perfectly rational if we assume that the previous people have relevant and independent knowledge. For example, they might have inside information about the value of the company. However, if everyone chooses to align, rather than making use of their own knowledge, then their decisions are no longer independent and the cascade can lead to the amplification of errors and to incorrect decisions.

Moreover, low-level mirroring of actions can also lead to disadvantages. In competitive games, such as rock–paper–scissors and matching pennies, people tend automatically to mirror the actions of their opponent. If you shape your hand into scissors, then I am more likely to do the same. This mirroring prevents players from achieving optimal performance [50,51]. Action mirroring can also interfere with performance in cooperative situations. For example, prior exposure to rhythmic auditory stimuli facilitates interpersonal motor coupling, but this can impede attainment of the dynamic coordination needed for joint task performance [52]. This is because, for many tasks, the appropriate actions for the individuals in the group should be complementary rather than aligned. A close analogy would be when a single person performs a bimanual task. The actions of the left and right hands will typically be complementary, although their movements are coordinated by a common representation of space and time.

Fusaroli & Tylén [53] have developed a quantitative approach enabling them to distinguish interactive alignment from interpersonal synergy (complementary pattern of behaviour). They found that while both forms of interaction were present in dialogue, only the synergistic aspects of dialogue predicted the level of collective performance.

Complementary actions are essentially forms of division of labour. Such divisions often lead to the emergence of a leader–follower relationship. For example, in the synchronized tapping studied by Konvalinka *et al.* [34] there are two task demands: first, to maintain the rhythm and, second, to stay in synchrony. An efficient division of labour is for one partner to maintain the rhythm while the other takes care of the synchrony [54]. In this case the partner who takes care of the synchrony has become the follower, as maintaining synchrony requires greater adaptation. Leader–follower relationships also emerge when division of labour gives one partner a more difficult task than the other. Vesper *et al.* [55] developed a synchronized jumping task in which two partners tried to synchronize the time at which their jumps landed, even when the partners had to jump different distances. When both partners had an easy jump they mutually adapted their timing. However, when one partner had a longer and therefore more difficult jump, the other partner took on a follower role and did all the adapting. A similar result was obtained by Skewes *et al.* [56] using a synchronized aiming task.

Leader–follower distinctions can also be observed in brain activity. EEG was measured from two participants while they performed the synchronized tapping task described above [57]. During the interaction there was a reduction in alpha power over frontal electrodes, but only in the participant who was the leader in terms of showing lower behavioural adaptation. This reduction presumably reflects the greater degree of prospective planning and control required for taking on the task of maintaining the beat.

In these examples, leaders are defined as adapting less than the followers. Another closely related aspect of leadership during joint action is that leaders can be defined as making their decision to act before the followers. For example, in pairs of homing pigeons, the more experienced pigeon makes navigational choices faster and becomes the leader [58]. A clear leader–follower distinction of this kind can solve many simple coordination problems. Coordination games, such as choosing whether to dodge left or right when meeting someone in a narrow passageway, are more likely to be correctly solved when one member of the pair, the leader, makes his move markedly earlier than the other [59].

### (d) Brain mechanisms for dynamic coordination

Our review of behavioural studies suggests that social interactions, including joint action, are often characterized by complementary action, rather than mirroring or alignment. Such complementary behaviour includes division of labour and the adoption of leader/follower roles. As yet the neural processes underpinning such behaviour have received little investigation.

The behavioural and conceptual analyses above highlight the need to go beyond measurement of phase synchrony (mirroring) in order to look at neural mechanisms underlying social interactions. One problem is that phase synchrony may reflect a common driving signal rather than brain-to-brain coupling. A greater limitation of the method, however, is that while phase synchrony between brain regions may reflect mirroring at various levels, this measure will not pick-up complementary behaviour. In this final section, we shall return to the study of communication, as this is a fundamental case for human social interaction. The shape of dynamic interaction across brains during communication is vastly richer than simple alignment or mirroring. Alignment is only one of the types of coupling that can link the sender's and the receiver's brains. The neural coupling framework can take the form of (i) alignment, when the receiver's neural patterns match those of the sender; (ii) conditional transformations, when the receiver's patterns reflect a predictable relation to the sender's neural patterns or (iii) synergies, when the activities of the two brains dynamically influence and constrain each other to optimize information sharing. We argue that, together, these three forms of dynamical coupling provide a means of transmitting information across brains.

Measuring a non-symmetric coupled dynamic is very challenging, however, and will require the development of new experimental settings and mathematical toolkits. C*onditional transformations* occur when the listener's neural pattern reflects a predictable transformation of the speaker's neural pattern. For example, the neural activity in a person being threatened is probably different than the activity in the person making the threat. The activity in the two individuals, however, may still possess a lawful relation, even if they do not mirror each other. We hypothesize that reliable transformations from the speaker's neural responses to the listener's neural responses can be characterized by inter-subject mutual information and transfer entropy analyses, adapted from information theory [60,61]. Mutual information allows us to measure the information shared between the two brains, while transfer entropy will allow us to measure the information transferred from the speaker to the listener [62].

Another type of neural coupling is *synergy,* which occurs when the activities of the individual brains dynamically influence and constrain each other to optimize information sharing. For example, in a successful dialogue, two interlocutors do not simply mirror each other, nor do they respond in fixed patterns. Rather, they continuously listen, cooperate, and adapt to one another [53]. This coordination reflects the tension between opposing tendencies in all forms of complex dynamical interaction: segregation versus coupling [60], which can be assessed using mathematical frameworks such as centre manifold theory [63].

Our studies of synchronized activity, when people watch the same film or hear the same story, reveal a hierarchy of brain activity [24]. At the lowest level there is common activation associated with perceptual processing, while at the highest level there is common activity associated with meaning. In parallel with these increasing levels of abstraction, there is a hierarchy of timescales [64], with the more abstract levels of representation being associated with longer integration time windows [62,65]. During communication there will be coupling between these various levels of representation, but the exact form of this coupling is likely to be different for different levels. At the highest level of representation there may be mirroring as the speakers achieve a mutual understanding, regardless of the exact set of words used to convey the ideas. Similarly, even at lower levels of the hierarchy the coupling may change as a function of task and context. For example, in order to check that mutual understanding has been achieved, we will often repeat back what has been said in different words. This would be an example of synergy, the dynamic adaptation and constraint through which minds can achieve shared understanding.

These considerations show that we need an approach to the analysis of brain-to-brain coupling that takes into account the hierarchical nature of the interaction and of the different time-scales appropriate at each of the different levels [62]. Hierarchical Bayesian analysis might be helpful here. This approach has been used to model visual perception in the brain [66] and also to model how people can infer the intentions of others [67]. There have been preliminary attempts to apply such modelling to the study of communication [68]. In order to support communication, the brain has to solve the hermeneutic problem: how to understand the intended meaning given a speaker's words [69]. From a Bayesian perspective, communication involves two agents trying to make inferences about each other's hidden internal states. This requires that the first brain must have a model of the second brain, which includes a model of the first brain—and so on *ad infinitum*. However, this infinite regress dissolves once both brains have a common model for predicting each other's behaviour. It is the emergence of this high-level mirroring, alignment and coupling during the interaction that grounds the interaction and makes human communication possible. So far, we have only sketches of the underlying mechanisms. The challenge for the study of brain-to-brain coupling is to develop detailed models of the dynamical interaction that can be applied at the behavioural levels and at the neural levels.
